# Amnion-Derived Teno-Inductive Secretomes: A Novel Approach to Foster Tendon Differentiation and Regeneration in an Ovine Model

**DOI:** 10.3389/fbioe.2021.649288

**Published:** 2021-03-11

**Authors:** Maria Rita Citeroni, Annunziata Mauro, Maria Camilla Ciardulli, Miriam Di Mattia, Mohammad El Khatib, Valentina Russo, Maura Turriani, Michael Santer, Giovanna Della Porta, Nicola Maffulli, Nicholas R. Forsyth, Barbara Barboni

**Affiliations:** ^1^Unit of Basic and Applied Biosciences, Faculty of Bioscience and Agro-Food and Environmental Technology, University of Teramo, Teramo, Italy; ^2^Department of Medicine, Surgery and Dentistry, University of Salerno, Salerno, Italy; ^3^School of Pharmacy and Bioengineering, Keele University School of Medicine, Stoke-on-Trent, United Kingdom; ^4^Research Centre for Biomaterials BIONAM, University of Salerno, Fisciano, Italy; ^5^Centre for Sports and Exercise Medicine, Barts and The London School of Medicine and Dentistry, Queen Mary University of London, London, United Kingdom

**Keywords:** amniotic stem cells, co-culture, conditioned media, tendon, tendon-differentiation, tissue engineering

## Abstract

Regenerative medicine has greatly progressed, but tendon regeneration mechanisms and robust *in vitro* tendon differentiation protocols remain to be elucidated. Recently, tendon explant co-culture (CO) has been proposed as an *in vitro* model to recapitulate the microenvironment driving tendon development and regeneration. Here, we explored standardized protocols for production and storage of bioactive tendon-derived secretomes with an evaluation of their teno-inductive effects on ovine amniotic epithelial cells (AECs). Teno-inductive soluble factors were released in culture-conditioned media (CM) only in response to active communication between tendon explants and stem cells (CM_CO_). Unsuccessful tenogenic differentiation in AECs was noted when exposed to CM collected from tendon explants (CM_FT_) only, whereas CM_CO_ upregulated *SCXB, COL I* and *TNMD* transcripts, in AECs, alongside stimulation of the development of mature 3D tendon-like structures enriched in TNMD and COL I extracellular matrix proteins. Furthermore, although the tenogenic effect on AECs was partially inhibited by freezing CM_CO_, this effect could be recovered by application of an *in vivo*-like physiological oxygen (2% O_2_) environment during AECs tenogenesis. Therefore, CM_CO_ can be considered as a waste tissue product with the potential to be used for the development of regenerative bio-inspired devices to innovate tissue engineering application to tendon differentiation and healing.

## Introduction

Tendon differentiation is a stepwise process characterized by the sequential expression of tissue specific markers, an *in vivo* occurrence through the interaction of inductive paracrine conditions and the recruitment of tissue progenitor stem cells (Brent, 2005; Nourissat et al., 2015; Citeroni et al., 2020). However, a comprehensive determination of the underlying tenogenic differentiation mechanisms remains elusive. To date, several strategies have been proposed using different stem cells sources and techniques, without reaching conclusive results (Govoni et al., 2017; Dale et al., 2018; Giai Via et al., 2018; Ciardulli et al., 2020b; Citeroni et al., 2020; Giordano et al., 2020).

The first challenge is the definition of a successful tendon differentiation process in the absence of consolidated markers. Indeed, the many genes linked to tendon development are in common with a wide range of other tissues, including muscle, bone, and cartilage (Liu et al., 2017). In addition, many of these genes are already expressed in mesenchyme-derived stem cell sources (Zarychta-Wiśniewska et al., 2019), making threshold level determination in tenogensis difficult. The most prevalent tenogenic markers are Scleraxis (SCX), Tenomodulin (TNMD), Collagen type I (COL I), Collagen type III (COL III), and Thrombospondin 4 (THSB4) (Ciardulli et al., 2020b; Citeroni et al., 2020). The transcription factor *SCX* is considered an early marker of tenogenesis, since it is active during development the early phase of progenitor cell commitment (Schweitzer et al., 2001; Brent, 2002). COL I and COL III are the major component of tendon ECM and their modulation is related to tendon homeostasis and healing (Maffulli et al., 2000; Sharma and Maffulli, 2005). TNMD and THBS4 are both considered late tendon markers: TNMD is abundantly expressed in the mature tissue (Docheva et al., 2005), while THBS4 contributes to the regulation of extracellular matrix deposition and in the repair of myotendinous junction (MTJs) (Frolova et al., 2014; Subramanian and Schilling, 2014). TNMD expression is positively regulated by *SCX* (Shukunami et al., 2006), and it is involved in tenocyte proliferation, aging, and in the formation of collagen fibrils (Docheva et al., 2005; Alberton et al., 2015). In fact, mice with loss of TNMD expression showed impaired tenocyte proliferation, reduced tenocyte density, and increased maximal and greater variation of fibril diameters (Docheva et al., 2005).

The partial comprehension of tendon differentiation underlying mechanisms operating, in particular, during adulthood has narrowed their exploitation in developing new cures and in designing innovative tissue engineering (TE) approaches (Bullough et al., 2008; Andia and Maffulli, 2017; Migliorini et al., 2020). Poor prognoses are currently related to tendinopathies as a consequence of the absence of an effective treatment and the poor spontaneous healing ability of the tissue from the high degree of specialization leading to a low cellularity and hypo-vascularity. These conditions appear responsible for the reparative processes activated in response to different tendon injuries leading to the formation of scar tissue (O'Brien, 1997; Sharma and Maffulli, 2005) that negatively affects tissue functionality. Indeed, tendon injuries remain at the frontier of advanced responses to health challenges and sectoral policy targets.

The reduced regenerative property is, however, a peculiarity of adult tendons. The ability of tendon to regenerate is strictly age-related (Stalling and Nicoll, 2008): fetal tendons, during the early and mid-gestational stages, regenerate efficiently after injuries (Holm-Pedersen and Viidik, 1972). The underlying mechanism remains to be clarified, but the ability of fetal tendons to activate regeneration without any scar deposition appears related to higher levels of fibroblast tendon-related gene expression (Tang et al., 2015) and to greater local paracrine activity (Russo et al., 2015). Indeed, elevated levels of key growth factors and cytokines are implicated in promoting the scarless phenotype (Liechty et al., 2000; Chen et al., 2005) with cellular migration and collagen production levels that may support fetal tendons elevated healing capabilities (Stalling and Nicoll, 2008; Russo et al., 2015). Approaching birth and during the post-natal lifetime, tendons undergo profound transformations by progressively losing cellularity and the capability to secrete growth factors (Ruzzini et al., 2014; Russo et al., 2015). Adult tendons, becoming differentiated structures, reduce their ability to activate regeneration process (Sharma and Maffulli, 2005, 2006; Wang, 2006) and, when injured, they repair exclusively through scar formation (Woo et al., 2000; Fenwick et al., 2002; Lin et al., 2004).

The hypothesis that alternate paracrine control exists between adult and fetal tendons is indirectly confirmed from *in vitro* experiments. Barboni et al. (2012a) demonstrated that Amniotic Epithelial Stem Cells (AECs) can be *in vitro* differentiated toward tendon-cell lineage when co-cultured with tendon explants (CO). However, the tendon explant ability in releasing inductive tenogenic soluble factors are strictly dependent on tendon origin. Fetal tendon explants were able to drive the differentiation of ovine AECs more efficiently than adult (Barboni et al., 2012a), resulting in increased tendon-related gene and protein expression. Moreover, AECs exposed to fetal tendon explants acquired a mature tenogenic phenotype organizing themselves in 3D tendon-like structures (Barboni et al., 2012a). Taking advantage from such a mechanism, tenogenic commitment was quickly induced in AECs seeded on an electrospun PLGA scaffold, increasing their expression of *TNMD* and COL I gene and protein (Russo et al., 2020).

This suggests that, similar to other 3D cell culture systems (Goers et al., 2014; Paschos et al., 2015; Jensen and Teng, 2020), tendon explants can be successfully exploited to recapitulate *in vitro* the dialogue between somatic and progenitor cell compartments as acted out during fetal tendon development. The teno-inductive process reported on AECs suggested that fetal tendon explants were able to activate *in vitro* the complexity of paracrine signaling controlling the differentiation and or regenerative outcomes (Citeroni et al., 2020).

Tendon explant-derived culture approaches may have different potential impacts. The production of an *ex vivo* model to reproduce key differentiation mechanisms (Barboni et al., 2012a) and the tissue engineering exploitation of a derived secretome enriched in teno-inductive compounds sit amongst these.

Starting from these premises, the culture conditions required to collect teno-inductive secretomes were validated by testing their biological effect. Indeed, the present research was aimed to define the differentiation action of conditioned media (CM) on AECs. In the absence of any molecular characterization, the process of *in vitro* tenogenesis was tested on an epithelial stem cells source and documented by studying tendon differentiation molecular end points. This rigorous approach has been adopted to standardize the more suitable protocol to collect teno-inductive CM and to define the cultural conditions to use them after storage. In detail, the first step was to compare the biological teno-inductive potential of CM collected from the fetal tendon explant cultured alone (FT) or from AECs co-cultured with tendon explants (CO) The bio-activity of CM were tested on AECs by evaluating their *in vitro* differentiation by testing late tendon-related genes and proteins expression. Then, once identified the CM displaying the greater teno-inductive properties, in the second step, the effects of freezing storage on CM were defined as *in vitro* proofs of concept for their small scale production and practical application of teno-inductive secretomes.

## Materials and Methods

### Ethics Statement

AECs cells and fetal tendon explants (FT), used within the study, were obtained from discarded tissues (fetuses and amniotic membranes of pregnant slaughterhoused animals) of feed chain animals by local slaughterhouse. For this reason, no ethic statements were required.

### Experimental Design

**Figure 1:**
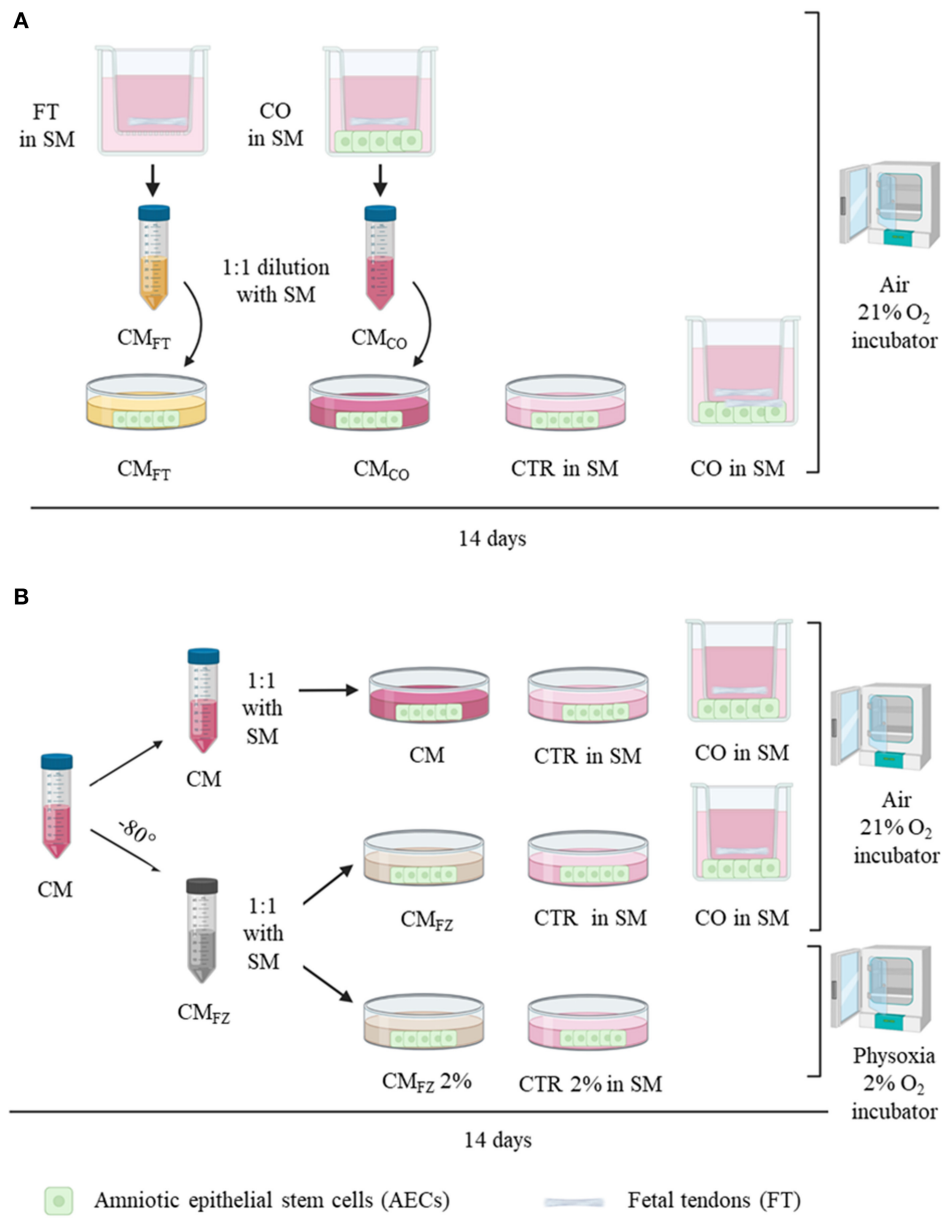
Experimental design. **(A)** Analysis of conditioned media (CM) derived from FT alone (CM_FT_) and from co-culture system (CM_CO_) on AECs cultured for 14 days prior to 1:1 dilution with standard media (SM) in air (21% O_2_) compared with AECs cultured alone (CTR) and in co-culture system (CO: FT+AECs). **(B)** Analysis of fresh collected CM after storage at −80°C (CM_FZ_) on AECs cultured in either air or and physoxia (2% O_2_) prior to 1:1 dilution with SM. FT, fetal tendons explants; AECs, amniotic epithelial stem cells. CO, co-culture with AECs and FT; CM_FT_, conditioned media of FT; CM_CO_, conditioned media of CO; CM_FZ_, CM after freezing thawing procedure; 21% O_2_, air oxygen incubator; 2% O_2_, incubator at 2%; SM, standard media.

The experimental design of this study is summarized in Figure 1. The first step of the experiment (Figure 1A) was designed to individuate the cell/tissue culture to maximize the *in vitro* release of bioactive secretomes including unknown teno-inductive soluble factors. To this aim, conditioned media (CM) were collected either from fetal tendon explants (CM_FT_) or from FT in co-culture (CO) with AECs (CM_CO_) and successively their biological teno-inductive properties were both evaluated on AECs. AECs incubated in Standard Media (SM) were used as a control (CTR) (Figure 1A). The second step of the experiment was performed to evaluate the impact of freeze (FZ) storage on the biological properties of CM (CM_FZ_) (Figure 1B) by comparing their teno-inductive influence on AECs with those of freshly isolated CM. Finally, the teno-inductive properties of CM_FZ_ were also analyzed on AECs cultured under air or physiological oxygen (physoxia) (Figure 1B: 21 vs. 2% O_2_, respectively). All cultures were maintained for 14 days in standard medium (SM) composed of α-MEM supplemented with 10% FBS (Gibco), 1 mL/100 mL L-glutamine and antibiotics/antimycotic solution (penicillin G sodium 100 U/ml, streptomycin 100 mg/ml, amphotericin B 0.25 mg/ml; Gibco, Invitrogen, Carlsbad, CA, USA) or CMs obtained by different culture conditions, and incubated at 38°C.

### AECs and Tendon Explant Collection

Fetuses of 25–35 cm of length at ~2–3 months of pregnancy (Barone and Edagricole, 1983), derived from 6 slaughtered animals, were used to obtains fetal tendons explants (FT) and amniotic membrane (AM) to isolate AECs.

AECs were collected as previously described (Canciello et al., 2018). In detail, the uterus wall was opened to collect AM under sterile conditions. Then, AM was mechanically peeled from the chorion with the aid of a stereomicroscope and dissected into 3–5 cm long fragments. Amnion pieces were washed in Phosphate Buffered Saline (PBS; Sigma Chemical Co. St. Louis, MO), and incubated in 0.25% Trypsin/EDTA 200 mg /L at 37.5°C for 20 min under gentle agitation. Cell suspensions were collected after filtration through a 40 μm cell filter and poured into a 50 mL Falcon tube, containing 10% Fetal bovine serum (FBS) (Gibco) to inactivate Trypsin, centrifuged, and the supernatant discarded. Concentration of vital cells were defined after pellet resuspension and Trypan Blue staining via a haemocytometer.

AECs were seeded immediately after isolation at a density of 3,000 cells/well in a 12 well-plate in SM and a flow cytometry phenotype characterization confirmed via negativity for haematopoetic markers (CD14, CD58, CD31, and CD45), positivity for surface adhesion molecules (CD29, CD49f, and CD166), and stemness markers (TERT, SOX2, OCT4, and NANOG), low expression for MHC class I molecules, and the absence of MHC class II (HLA-DR) antigens, as previously reported (Barboni et al., 2012a,b). FT were isolated from the forefeet and the peritendineum removed under sterile conditions (Barboni et al., 2012a). Small pieces of fresh tendon, about 3 mm in size, were isolated, and mechanically disaggregated under a stereomicroscope with forceps to maximize the interface between soft tissue and medium. FT explants were washed twice in PBS with 1% antibiotics and equilibrated in SM at room temperature (RT) for 10 min before transference into an incubator.

### Transwell FT and AECs Co-cultures

AECs co-culture (CO) with FT was performed using a transwell system as previously described (Barboni et al., 2012a). The AECs were plated onto 12 well-plates at 3,000 cells/well in SM. Transwell chambers (pore size 0.4 μm; Costar, NY, USA), containing FT explants (*n*: 2 FT for1 mm^3^ in size/trans-well), were inserted into the wells and cultured for FT cultures and FT+AECs co-cultures, both incubated in air supplemented with 5% CO_2_ at 38°C for 14 days. Each experiments were performed in triplicate (*n* = 3 experimental replicates) by comparing cells derived from 6 different fetuses (*n* = 6 biological replicates). The CM were collected at each medium change and processed as described below.

### Conditioned Media Collection, Storage and Use

CM from co-culture (CM_CO_) and from FT (CM_FT_) were collected every 2 days, centrifuged at 300 × g to eliminate cell debris and used for further experimentation. Immediately after collection freshly isolated CM_CO_ and CM_FT_, were diluted 1:1 in SM (Alves da Silva et al., 2015) and tested on AECs culture for their biological teno-inductive influence over 14 days of incubation (Figure 1A), and compared with the direct effect of CO as well as with AECs alone incubated in SM (CTR).

The CM tenogenic effect was also tested after freezing. To this aim, CM were stored from at least 1 month to maximum 3 months at −80°C (CM_FZ_). After thawing in 37°C bath, they were used (1:1 dilution in SM) on AECs, and assessed for their bioactivity in comparison with freshly collected CM. CM_FZ_ bioactivity was evaluated on AECs incubated in 2% O_2_ tension, thus mimicking the *in vivo* physiological condition of tendon tissue (physoxia) (Benjamin and Ralphs, 1997; Sharma and Maffulli, 2005; Shukunami et al., 2008) or in air oxygen by using conventional *in vitro* conditions (air; Figure 1B). In both the experimental groups, CM_FZ_ were replaced every 2 days. AECs without CM were used as internal negative control (CTR).

### Morphological Evaluation of Teno-Inductive Effect on AECs

AECs morphology evaluation was performed using inverted microscopy according to previously described criteria (Barboni et al., 2012a). In detail, at the end of the culture period, the structures present in each well of every different experimental condition were cataloged into three different 3D cell aggregate types: circular aggregates, elongated structures, and 3D tendon-like structures. All structures in each well were counted. Data were obtained at least from six different biological replicates (*n* = 6 animals) performed in triplicate (*n* = 3 experimental replicates), and reported as the mean ± S.D.

### Total RNA Isolation and RT-qPCR

Genes related to epithelial mesenchymal transition (EMT), oxygen signaling response, and tenogenic differentiation were examined on whole lysates from AECs isolated after 14 days of culture in different experimental conditions. *SNAIL, TWIST*, and *VIM* genes were considered as EMT-related markers, *HIF 1*α to test oxygen signaling response, and *SCXB, COL I, COL III, TNMD, THSB4* as tendon-related genes (see Table 1). AECs freshly isolated from the membrane immediately stored in liquid nitrogen (T0) were used as baseline expression control. In detail, total mRNA was extracted by RNeasy Mini Kit (Qiagen), according to the manufacturer instructions. Total RNA integrity was evaluated by 1% agarose gel electrophoresis with GelRed staining (Biotium). Quantification of total RNA samples was assessed by using Thermo Scientific NanoDrop 2000c UV-Vis spectrophotometer at 260 nm. Digestion of genomic DNA was carried out by DNaseI (Sigma) exposing the samples for 15 min at RT. One step Real-time qPCR analysis was performed with 10 ng of total mRNA by using SensiFAST^TM^ SYBR Lo-ROX One step kit (Bioline) and the gene primers in Table 1. The reactions were carried out with 7500 Fast Real-time PCR System (Life Technologies) by using the two-step cycling protocol for 40 cycles (5 s at 95°C for denaturation and 30 s at 60°C for annealing/extension) followed by melt-profile analysis (7500 Software v2.3). For each gene analyzed, each sample was performed in triplicate, and values were normalized to endogenous reference gene GAPDH. The relative expression of different amplicons was calculated by the comparative Ct (ΔΔCt) method, converted to relative expression ratio (2^−ΔΔCt^) (Livak and Schmittgen, 2001), and expressed as fold change over AECs T0 = 1. For primers sequences, see Table 1.

**Table 1:** Primers details used for RT-qPCR analysis.

**Gene**	**Accession number**	**Sequences**	**Product size (bp)**
*VIM*^a^	XM_004014247.4	For: 5′-GACCAGCTCACCAACGACA-3′	93
		Rev: 5′-CTCCTCCTGCAACTTCTCCC-3′	
*SNAIL*^a^	XM_004014881.2	For:5′- GTCGTGGGTGGAGAGCTTTG−3′	119
		Rev: 5′- TGCTGGAAAGTGAGCTCTGG−3′	
*TWIST*^a^	XM_004008211.4	For: 5′-GCCGGAGACCTAGATGTCATTG-3′	150
		Rev: 5′-CCACGCCCTGTTTCTTTGAAT-3′	
*TNMD*^b^	NM_001099948.1	For: 5′-TGGTGAAGACCTTCACTTTCC-3′	352
		Rev: 5′-TTAAACCCTCCCCAGCATGC-3′	
*SCXB*^b^	XM_866422.2	For: 5′-AACAGCGTGAACACGGCTTTC-3′	299
		Rev: 5′-TTTCTCTGGTTGCTGAGGCAG-3′	
*COL I*^b^	AF129287.1	For: 5′-CGTGATCTGCGACGAACTTAA-3′	212
		Rev: 5′-GTCCAGGAAGTCCAGGTTGT-3′	
*COL III*^b^	AY091605.1	For: 5′-AAGGGCAGGGAACAACTTGAT-3′	355
		Rev: 5′-GTGGGCAAACTGCACAACATT	
*THSB4*^b^	NM_001034728.1	For: 5′-CCGCAGGTCTTTGACCTTCT-3′	231
		Rev: 5′-CAGGTAACGGAGGATGGCTTT-3′	
*HIF 1α*	XM_027971913.1	For: 5′-TGCTCATCAGTTGCCACTTC-3′	311
		Rev: 5′-TTTCCTCATGGTCACATGGAT-3′	
*GAPDH*^b^	AF030943.1	For: 5′-CCTGCACCACCAACTGCTTG-3′	224
		Rev: 5′-TTGAGCTCAGGGATGACCTTG-3′	

Primers used in

a Canciello et al. (2017)

and

b *Barboni et al. (2012a)*.

### Total Protein Extraction and Western Blotting

The late tendon marker TNMD and the HIF 1α inducible factor were both analyzed at protein level by using WB assay. To this aim, total protein was extracted from each sample in lysis buffer (50 mM Tris HCl pH 8, 250 mM NaCl, 5 mM EDTA, 0,1% Triton X-100 10%) with Phosphatase Inhibitor (P5726, Sigma) and Protease Inhibitor Cocktails (P8340, Sigma) diluted according to manufacturing instruction. Samples were put on ice for 30 min, and then centrifuged at 12,000 × g for 10 min at 4°C. The supernatant was collected, and 1 μL used to determine protein concentration with Quick Start^™^ Bradford 1x Dye Reagent (BioRad). Afterwards, 30 μg of total protein was separated by 10% SDS-PAGE, and then transferred to nitrocellulose membranes (Millipore, Bedford, MA) with TURBO Transfers (BioRad). Membranes were subsequently incubated with 5% non-fat dry milk (Sigma) in 0.1% (v/v) Tween 20 in Tris-buffered saline (T-PBS) for 1 h, at 4°C. Primary antibodies against mouse-TNMD, mouse -HIF 1α, and rabbit-αTUBULIN proteins (see Table 2) were incubated overnight according to manufacturer instructions. Finally, membranes were incubated with specific secondary HRP conjugated IgG antibodies for 1 h, at room temperature. Protein bands were visualized by Euroclone ECL reagents (LiteAblot PLUS Euroclone EMP011005) and detected by Azure Byosystem. Densitometric analysis was performed using Image Lab software (version 4.0, Biorad). Relative protein expression values were normalized to the corresponding Tubulin expression. Antibodies details are shown in Table 2.

**Table 2:** Details of primary and secondary antibodies used for Western Blot Analysis.

**Primary antibody**	**Conc. μg/μL**	**Secondary antibody**	**Conc. μg/μL**
Mouse TUBULIN (SiSMa T5168)	0.5	Anti-mouse HRP conjugated (Santa Cruz sc 516102)	0.04
Rabbit TNMD (Abcam ab81328)	0.5	Anti-rabbit HRP conjugated (Santa Cruz sc 2357)	0.04
Mouse HIF 1α (NovusBio NB 100-123)	2	Anti-mouse HRP conjugated (Santa Cruz sc 516102)	0.2

### Immunohistochemistry

TNMD and COL I protein expression and localization were recorded on *in vitro* cultured AECs by immunohistochemistry (IHC). In detail, cells were fixed in 4% paraformaldehyde/PBS for 10 min. After 3X 5 min PBS washes, the cells were permeabilised with 0.1% Triton X-100/PBS for 10 min at RT for TNMD immunostaining, or with Tween 20–0.05%/ BSA 1%/ PBS for COL I immunostaining procedures. Blocking was performed by incubating cells at RT in PBS/1% BSA for 1 h. Cells were incubated with rabbit TNMD (10 μg/μL; Biorbyt, Cambridge, UK) and mouse COL I (10 μg/μl; EMD Millipore Corporation, Temecula, USA) primary antibodies overnight at 4°C. Anti-rabbit Cy3 conjugated (3.75 μg/μL; Millipore, Temecula, USA) or Anti-mouse 488 FITC conjugated (1 μg/μl; Bethyl, Montgomery, USA) secondary Abs were then used for 1 h at RT. The omission of primary antibodies (Abs) were used as negative controls of reactions. Cell nuclei were identified with DAPI counterstaining. Morphometric evaluation of the images was obtained using Axioskop 2 Plus incident light fluorescence microscope (Carl Zeiss, Oberkochen, Germany) equipped with a CCD camera (Axiovision Cam, Carl Zeiss) with a resolution of 1300 × 1030 pixels, configured for fluorescence microscopy, and interfaced to a computer workstation, provided with an interactive and automatic image analyser (Axiovision, Carl Zeiss). For each experimental condition, the reaction was performed in triplicate (*n* = 3) on each biological replicates (*n* = 6 animals).

### Cell Orientation Analysis

Cells orientation of AECs cells after 14 days of culture with CM_CO_ and CM_FZ_ under air and physoxia condition was assessed on the obtained 3D tendon-like structures using the Directionality Plugin of ImageJ (Sensini et al., 2018; El Khatib et al., 2020), to better determine the teno-inductive properties of CMs. Briefly, Plugin chops the IHC images with cells nuclei DAPI counterstained into square pieces and computes their Fourier power spectra allowing the generation of statistics data on the basis of the highest peak found represented by direction (the center of the Gaussian) and dispersion (the standard deviation of the Gaussian). Images of healthy tendon explants were used as internal control to establish the reference values for the analyses. This approach quantifies the cells direction, dispersion, and amount (the sum of the histogram from center-S.D. to center ± S.D, divided by the total sum of the histogram). The real histogram values are used for the summation, not the Gaussian fit (amount). A direction of 0 degree means that the cells are oriented with the longitudinal axis of the sample. The higher is the dispersion value, the lower is the homogeneity of cells orientation.

Statistical analyses were performed by One Way ANOVA and expressed as means ± S.D of six biological replicates (*n* = 6 animals)/each experimental condition in triplicate.

### Statistical Analysis

The quantitative data of the analysis were obtained by analyzing at least three samples for each experimental condition performed in triplicate (*n* = 3) on each biological replicates (*n* = 6 animals). The RT-qPCR results were firstly assessed for distribution using Shapiro Wilks test. Data sets were compared using Kruskal-Wallis test followed by Dunn's *post hoc* test. The quantitative data for morphological structures, WB analysis and cells orientation, were expressed as mean ± S.D by using One Way ANOVA followed by Tukey *post hoc* test (GraphPad Prism 6, GraphPad Software, San Diego, CA, USA). Significant was set at *p* < 0.05.

## Results

### CM_CO_ Displayed an Enhanced Teno-Inductive Influence on AECs

**Figure 2:**
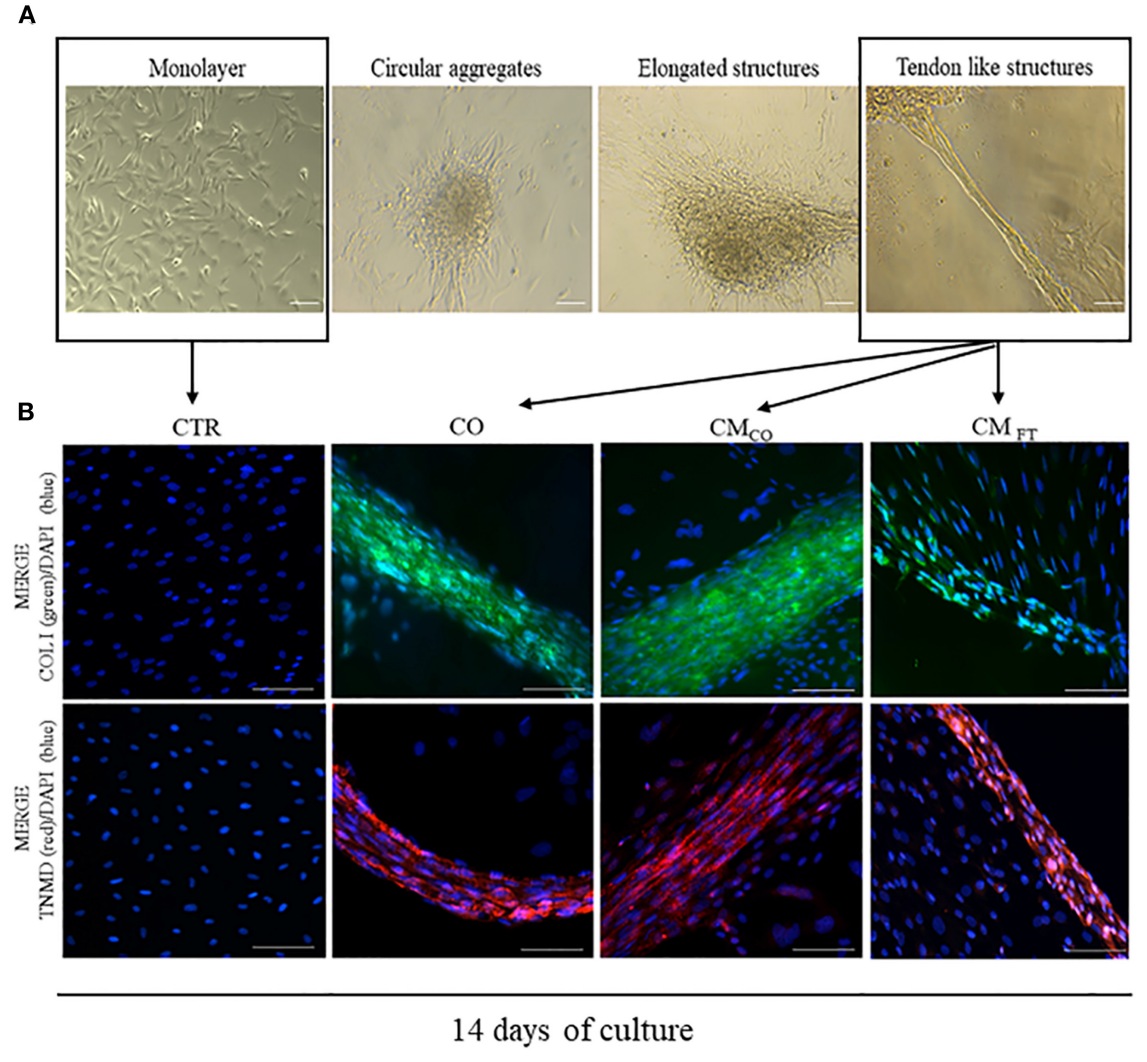
**(A)** Representative images of different cell aggregates developed by AEC cultured under CTR and different teno-inductive (CO and CM) cultural conditions. Images were obtained with Olympus microscope IX-50 and processed with Image-Pro Insight software. Magnification 10X, scale bar 100 μm. **(B)** Representative immunofluorescent images displaying the main cell organization after 14 days of culture as CTR, CO, CM_CO._ and CM_FT_, respectively. AEC under CTR condition after 14 days lead a confluent monolayer negative for both COL I and TNMD. On the contrary, CO, CM_CO_, and CM_FT_ promote formation of COL I and TNMD positive 3D structures even if only in CO and CM_CO_ both the proteins reach a high concentration combined with an extracellular localization. Representative IHC images of COL I (green color) and TNMD (red color) protein expression in AECs after 14 days of culture under different experimental conditions. Nuclei were counterstained with DAPI (blue color). Magnification 20X, scale bar: 100 μm.

CM derived from FT (CM_FT_) and CO (CM_CO_) were evaluated in their biological teno-induction capacity on AECs differentiation into tendon lineage over 14 days of culture. Specifically, AECs, following on from incubation, may acquire different phenotypes ranging from a persistent planar, monolayer, organization to 3D cellular aggregates with circular or elongated structures and ultimately tendon-like units. When AECs are exposed to CM, they first assumed a sheet monolayer in few days before spontaneously forming cell clusters. Sometimes they developed circular aggregates, randomly distributed within the well, which, at the end of the incubation period, may morph into elongated structures with different degree of organization. The more complex elongated structures acquired a 3D architecture, at the end of incubation, by detaching from the monolayer substratum and maintaining peripheral contacts with the well-border, becoming 3D tendon-like units at day 14. The 3D tendon-like structures reached a final size ranging from 0.4 to 2 mm in length without any difference amongst the groups (data not shown). These main AEC phenotypes obtained during the 14 days of culture, are represented in Figure 2A. CM_CO_ displayed a higher ability to promote an AECs morphology shift toward 3D tendon-like structure organization accordingly to the incidence of 3D tendon-like structures (Table 3) and their proteins composition (Figure 2B).

**Table 3:** CM_CO_ promotes the formation of elongated and tendon-like structures.

**Culture conditions**	**Mean of the number of 3D cells structures/well±** **S.D**		
	**Circular aggregates**	**Elongated structures**	**Tendon-like structures**
CTR	0	0	0
CO	0	2 ± 0.53^*^	11 ± 0.83^*^^b^^c^
CM_CO_	0	3 ± 0.71^*^	8 ± 0.86^*^^c^
CM_FT_	6 ± 0.98^*^^a^^b^	4 ± 0.75^*^^a^	2 ± 0.78^*^

Number of 3D cells structures counted in different experimental conditions and expressed as the mean ± S.D obtained by analyzing 12 well/ experimental group for each animal (n = 6). Data were analyzed by One Way ANOVA Test followed by Tuckey post hoc test. Statistics was performed on the same typology of structure between the experimental groups. Values were considered statistically significant for p < 0.05 for superscript

* *vs. CTR*;

a *vs. CO*;

b *vs. CM_CO_*;

c *vs. CM_FT_*.

The highest prevalence of 3D tendon-like structures was recorded in AECs exposed to CO and in CM_CO_ (both *p* < 0.05 vs. CM_FT_). Conversely, CM_FT_ induced in AECs a significant high percentage of circular aggregates (*p* < 0.05 vs. CO and CM_CO_, respectively) and elongated structures (*p* < 0.05 vs. CO).

In this experimental phase, IHC was carried out in order to evaluate the presence and localization of COL I and TNMD tenogenic proteins, representing end point signals of tendon like cells.

Exclusively CO, CM_CO_ and were able to switched on the expression of COL I and TNMD that is absent in freshly isolated AEC (data not shown) and in AEC cultured for 14 days under CTR condition. However, IHC results indicated that the process of teno-differentiation was more evident in both CO and CM_CO_ (Figure 2B) where both COL I and TNMD seem to reach higher levels of expression but, above all, they did not have an exclusive cytoplasmic localization but also they appeared as a components of extracellular matrix.

In fact, AECs exposed to CO or to CM_CO_ revealed an advanced cellular and extracellular matrix (ECM) in the 3D structures. The tendon-related phenotype changes, at both morphological and molecular levels, were promoted in epithelial cells derived from amniotic membrane exclusively in response to inductive cultural conditions. Indeed, AECs did not express COL I and TNMD either immediately after isolation (data not shown) or at the end of culture when they were maintained under CTR conditions (Figure 2B). Instead, COL I appeared in 3D circular aggregates (data not shown) while TNMD was mainly in tendon-like structures (Figure 2B). The qualitative expression of both the proteins was, particularly, elevated in CO and CM_CO_ derived 3D tendon-like structures, when COL I and TNMD proteins were mostly localized along the 3D structures and not in the other dispersed cells (Figure 2B)_._ In addition, the 3D tendon-like structures of CO and CM_CO_ showed a more advanced tendon-like organization with elongated cells displaying nuclei mainly oriented along the major axis of the *in vitro* developed tissue units (Figure 2B, CO and CM_CO_). By contrast, the 3D tendon-like structures induced by CM_FT_ (Figure 2B, CM_FT_) were thinner with a lesser degree of tissue organization and faint fluorescent signal revealing weak expression of COL I and TNMD principally expressed at an intracellular level (Figure 2B, CM_FT_).

**Figure 3:**
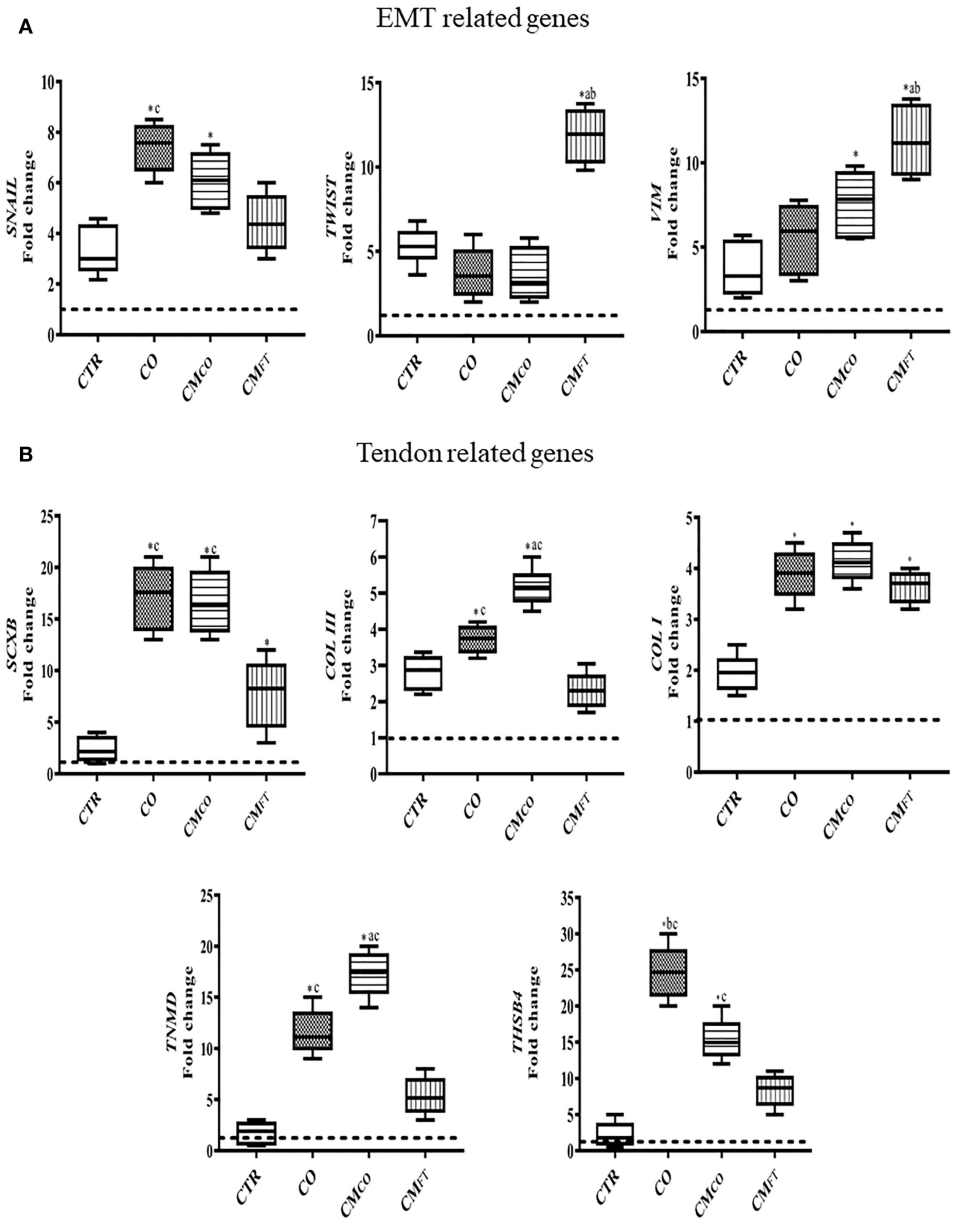
CM_CO_ promotes upregulation of tendon-linked gene expression. Gene expression profile by Real time qPCR analysis of **(A)** EMT genes and **(B)** tendon related genes in AECs cells cultured for 14 days in different experimental conditions. Relative quantification of each mRNA gene expression normalized to endogenous GAPDH (internal control) was calculated using the ΔΔCt method and expressed as fold change over the AECs T0 =1 (calibrator; dashed line). Values were considered significant for *p* < 0.05, with the indicated superscripts ^*^vs. CTR, ^a^vs. CO, ^b^vs. CM_CO_, ^c^vs. CM_FT_, respectively.

**Figure 4:**
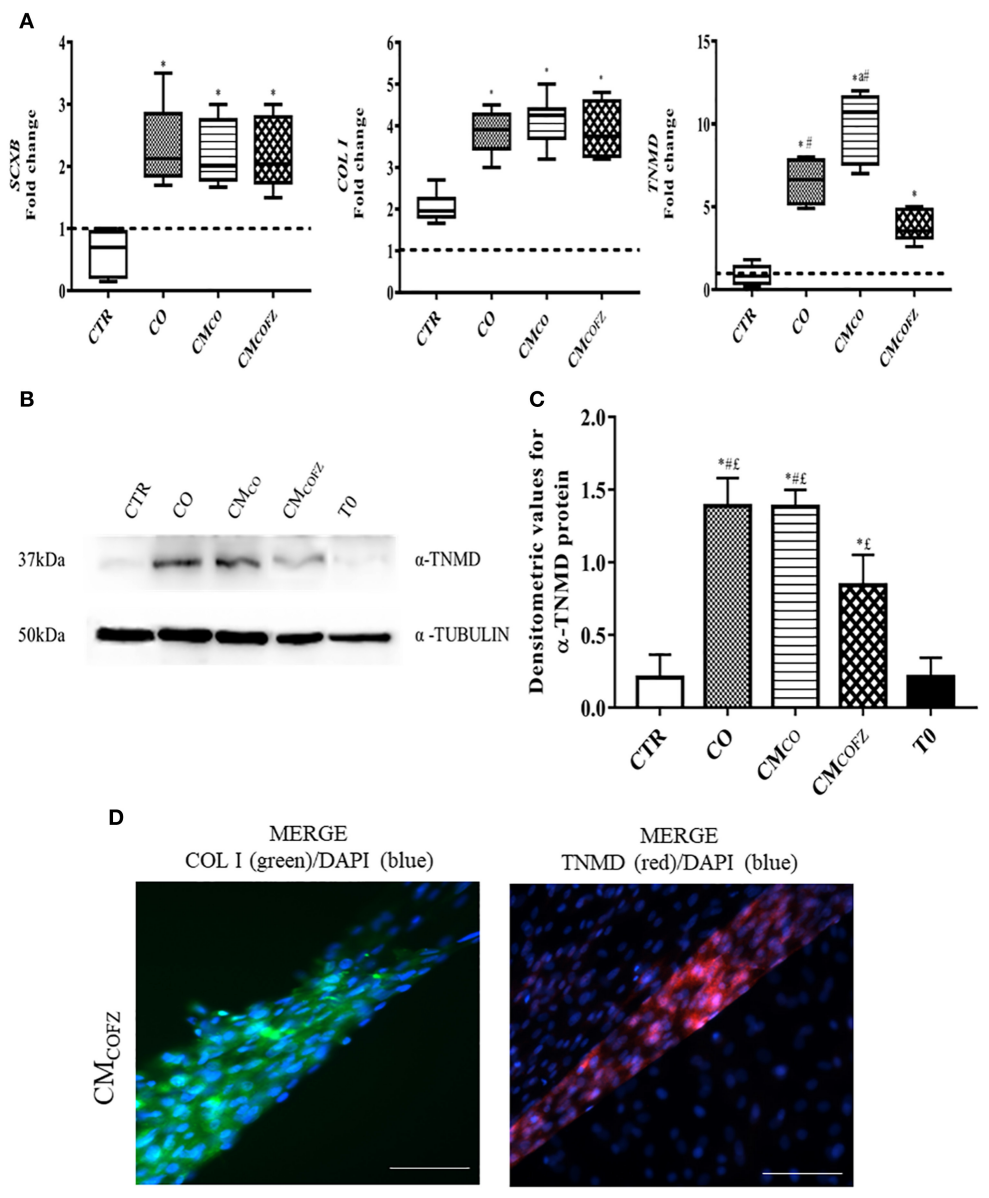
Evaluation of tenogenic potential CM_COFZ_ on AECs after 14 days of culture. **(A)** Real time qPCR analysis for *SCXB, COL I*, and *TNMD* tendon related genes in AECs cells cultured for 14 days in different experimental conditions. Relative quantification of each mRNA gene expression normalized to endogenous GAPDH (internal control) was calculated using the ΔΔCt method and presented as fold change over the AECs T0 =1 (calibrator; dashed line). Values were considered significant when *p* < 0.05, with superscripts ^*^vs. CTR, ^a^vs. CO, ^#^vs. CM_COFZ_. **(B)** Representative Western Blot image for α-TNMD and α-TUBULIN proteins expression on different experimental conditions. **(C)** α-TNMD protein quantification normalized on α-TUBULIN expression levels and expressed as means ± S.D of three replicates/each experimental condition. Values were considered significant when p < 0.05, with the indicated superscripts ^*^vs. CTR, ^#^vs. CM_COFZ_,^£^vs. T0. **(D)** Representative IHC images for COL I (green color) and TNMD (red color) protein expression in AECs after 14 days of culture under CM_COFZ_ experimental condition. Nuclei were counterstained with DAPI (blue color). Magnification 20X, scale bar: 100 μm.

The tendon-like AECs differentiation outcome induced by CM_CO_ was confirmed by gene expression analysis (Figure 3). Tendon-related gene expression was predominantly upregulated in CO and CM_CO_ treated-samples, while CM_FT_ mainly induced overexpression of EMT-linked transcripts (Figures 3A,B). In detail, CM_FT_ displayed a limited up-regulatory influence on EMT transcription factor *TWIST* (*p* < 0.05 vs. CTR, CO, and CM_CO_) and EMT endpoint marker *VIM* (*p* < 0.05 vs. CTR, CO, and CM_CO_) and two tendon-related genes *SCXB* and *COL I* (both *p* < 0.05 vs. CTR) (Figure 4B). On the contrary, a more advanced tendon expression profile was observed in AECs exposed to CO and CM_CO_. The early and late tendon-related genes, *SCXB* and *THSB4* and *TNMD*, respectively, showed a significant upregulation in AECs under both CO and CM_CO_ conditions (both *p* < 0.05 vs. CM_FT_) while *COL III* reached the highest levels of expression in CM_CO_-treated AECs (*p* < 0.05 vs. CO and CM_FT_) (Figure 3B).

According to the biological responses, of both genes profile expression and morphological phenotype, induced *in vitro* on AECs by the derived secretomes, CM_CO_ was selected for its higher teno-inductive performance and used for the following experiments.

### Freezing Reduced the Tenogenic Induced Activities of CM_CO_

To extend the impact of CM_CO_ on *in vitro* tenogenesis and in cell-free regenerative medicine innovation, the viability of CM_CO_ storage by freezing was evaluated. The teno-inductive potential of CM_CO_ stored at −80°C (CM_COFZ_) was compared with those of CO and freshly collected CM_CO_ in their effect on *in vitro* AECs tendon differentiation (Figure 1B). A reduction in the tendon-inductive action of CM_COFZ_ was suggested by the morphological outcomes of the *in vitro* cultures (Table 4 and Figure 4D) and confirmed by the tendon-related gene profiles (Figure 4A), respectively. AECs treated with CM_COFZ_ displayed a reduced ability to induce the development of 3D structures (*p* < 0.05 vs. both CO and CM_CO_). AECs in CM_COFZ_ aggregated mainly circular (*p* < 0.05 vs. both CO and CM_COFZ_) and elongated structures (*p* < 0.05 vs. CO and CM_CO_) with a limited number of 3D structures that reached the tendon-like stage (Table 4).

**Table 4:** CM_COFZ_ has a reduced teno-inductive capacity.

**Culture conditions**	**Mean of the number of 3D cells structures/well±** **S.D**		
	**Circular aggregates**	**Elongated structures**	**Tendon-like structures**
CTR	0	0	0
CO	0	2.5 ± 0.53^*^	12 ± 0.83^*^^b^^#^
CM_CO_	0	2.6 ± 0.71^*^	7.5 ± 0.86^*^^#^
CM_COFZ_	4 ± 0.98^*^^a^^b^	5.5 ± 0.75^*^^a^^b^	3 ± 0.78^*^

Number of 3D cells structures counted in different experimental condition and expressed as the mean ± S.D obtained by analyzing 12 well/ experimental group for each animal (n = 6). Data were analyzed by One Way ANOVA Test followed by Tuckey post hoc test. Statistics was performed on the same typology of structure between the experimental groups. Values were considered statistically significant for p < 0.05 for superscript

* *vs. CTR*,

a *vs. CO*,

b *vs. CM_CO_*,

# *vs. CM_COFZ_*.

The gene profile of AECs exposed to CM_COFZ_ confirmed their reduced stage of tendon differentiation. Indeed, the CM_COFZ_ induced AECs showed upregulation exclusively of *SCXB* and *COL I* that reached expression levels similar to those recorded under CO and CM_CO_ conditions (*p* < 0.05 vs. CTR). By contrast, the late marker of tenogenesis *TNMD* never reached the levels recorded in CO and CM_CO_ structures (for both *p* < 0.05; Figure 4A). The IHC analysis (Figure 4D) revealed that the rare 3D tendon-like structures obtained under CM_COFZ_ condition displayed the positivity for both COL I and TNMD proteins involving either the intracellular or extracellular distribution (Figure 4D). However, the protein content quantified by using Western Blot analysis (Figures 4B,C), confirmed a significant TNMD protein levels reduction in CM_COFZ_ respect to CO and CM_CO_ (for both *p* < 0.05), even if it was overexpressed respect to the CTR and T0 samples (for both *p* < 0.05) (Figure 4C).

### Physoxia Restored the Tenogenic Potential of Frozen Co-culture Conditioned Media

**Figure 5:**
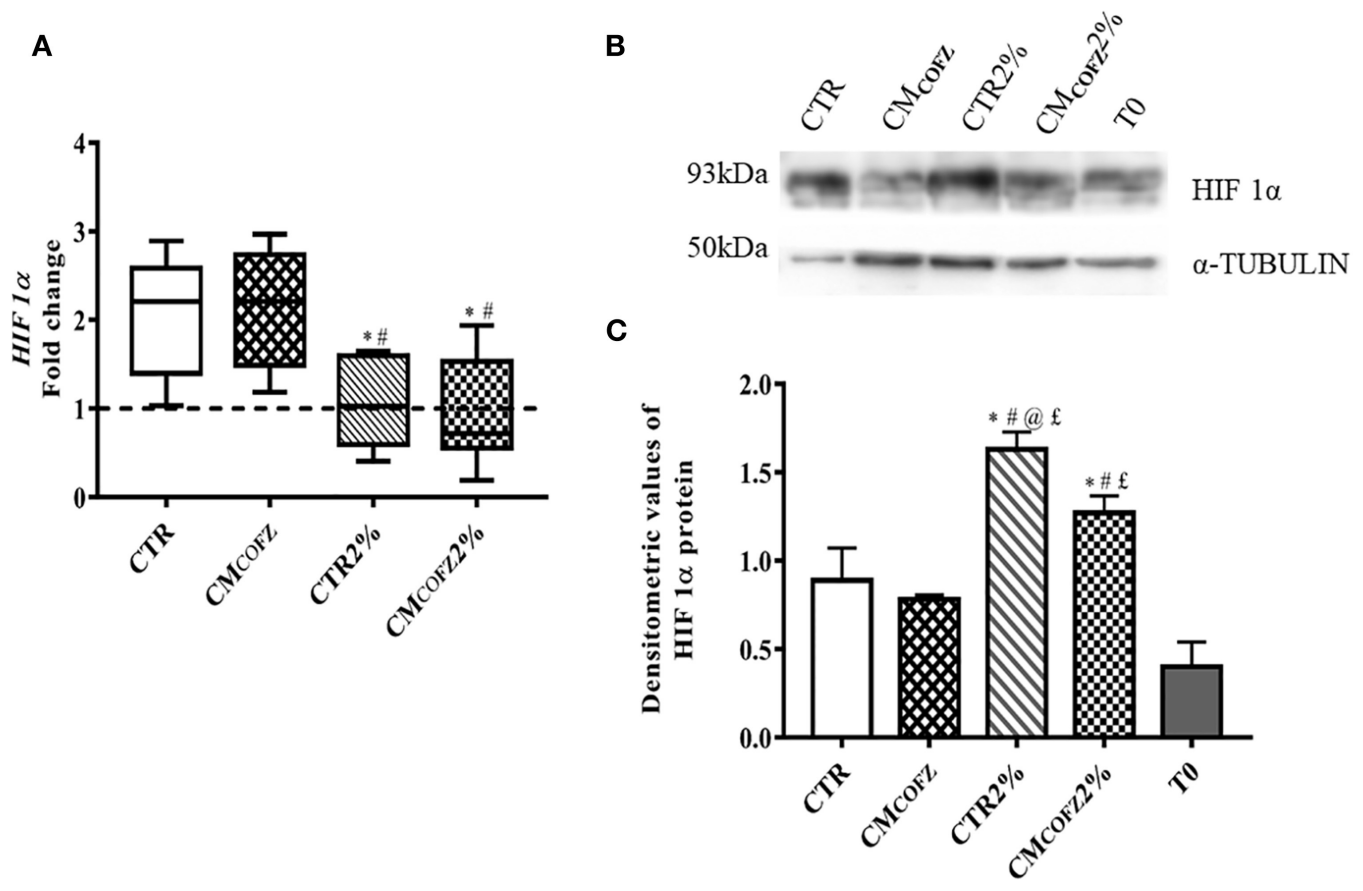
Physoxia effects of tenogenic potential of CM_COFZ_ on AECs after 14 days of culture. **(A)** Real time qPCR analysis for *HIF 1*α expression in normoxia CTR and CM_COFZ_ and in physoxia CTR 2% and CM_COFZ_ 2% conditions. Relative quantification of each mRNA gene expression normalized to endogenous GAPDH (internal control) was calculated using the ΔΔCt method and presented as fold change over the AECs T0 =1 (calibrator; dashed line). **(B)** Representative Western Blot image for HIF 1α and α-TUBULIN proteins expression on different experimental conditions. **(C)** HIF 1α protein quantification normalized on α-TUBULIN expression levels and expressed as means ± S.D of three replicates/each experimental condition. Values were considered significant when *p* < 0.05, with the indicated superscripts ^*^vs. CTR, ^#^vs. CM_COFZ_, ^@^vs. CM_COFZ_ 2%,^£^vs. T0.

The culture in 2% O_2_ was able to influence the phenotype of AECs and to enhance the teno-inductive action of CM_COFZ_. The effect of O_2_ tension on AECs cultures was confirmed by the expression of the physoxia marker *HIF 1*α analyzed at gene and protein level (Figure 5). More in detail, the expression *HIF 1*α were significantly downregulated in samples under physoxia (*p* < 0.05 both CTR 2% and CM_COFZ_ 2% vs. CTR and CM_COFZ_) (Figure 5A). Where, on the contrary, a significant higher levels of HIF 1α protein was detected (*p* < 0.05 of both CTR 2% and CM_COFZ_ 2% vs. CTR and CM_COFZ_) (Figures 5B,C).

Chronic exposure of AECs to physoxia had an interestingly effect on their phenotype (see Table 5). In particular, physoxia increased the ability of AECs exposed to CM_COFZ_ to develop *in vitro* 3D tendon-like structures (*p* < 0.05 of CM_COFZ_ in 2% O_2_ vs. CM_COFZ_) (Table 5).

**Table 5:** Physoxia recovers CM_COFZ_ teno-inductive capacity.

**Culture conditions**	**Mean of the number of 3D cells structures/well±** **S.D**		
	**Circular aggregates**	**Elongated structures**	**Tendon-like structures**
CTR	0	0	0
CM_COFZ_	2.5 ± 0.96^*^^@^	5 ± 0.22^*^^§^^@^	4.5 ± 0.65^*^^§^
CTR 2%	3 ± 0.89^*^^@^	2 ± 0.75^*^	0
CM_COFZ_ 2%	1 ± 0.54	3 ± 0.77^*^	6.5 ± 0.72^*^^#^^§^

Number of 3D cells structures counted in different experimental condition and expressed as the mean ± S.D obtained by analyzing 12 well/ experimental group for each animal (n = 6). Data were analyzed by One Way ANOVA Test followed by Tuckey post hoc test. Statistics was performed on the same typology of structure between the experimental groups. Values were considered statistically significant when p < 0.05 for superscript

* *vs. CTR*,

# *vs. CM_COFZ_*,

§ vs. CTR 2%

@ *vs. CM_COFZ_ 2%*.

**Figure 6:**
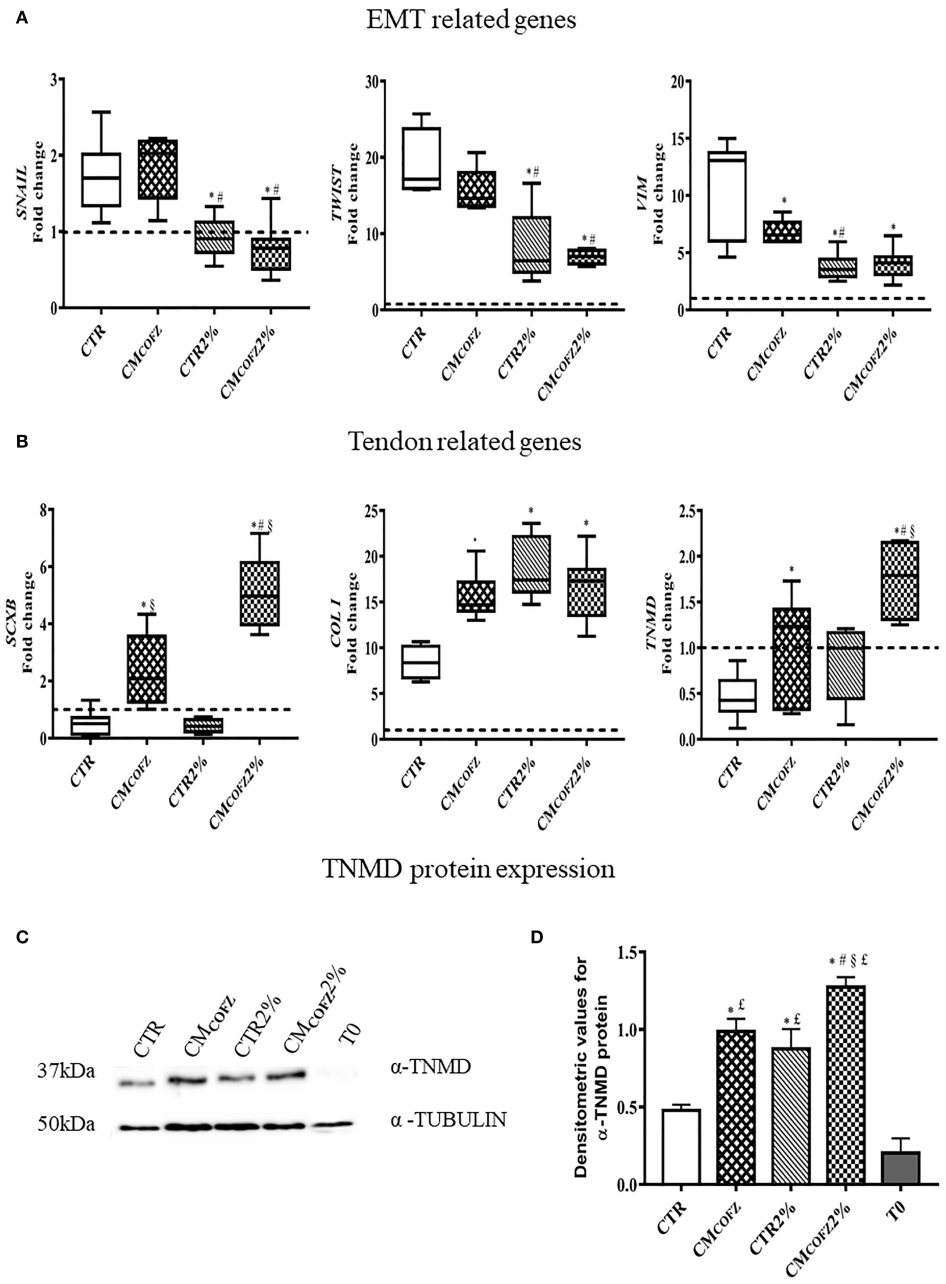
Evaluation of tenogenic potential of CM_COFZ_ 2%. Real time qPCR analysis for **(A)** EMT genes and **(B)** tendon related genes on samples in AECs cells cultured for 14 days in different experimental conditions. Relative quantification of each mRNA gene expression normalized to endogenous GAPDH (internal control) was calculated using the ΔΔCt method and presented as fold change over the AECs T0 =1 (calibrator; dashed line). **(C)** Representative Western Blot image for α-TNMD and α-TUBULIN proteins expression. **(D)** α-TNMD protein quantification normalized on α-TUBULIN expression levels and expressed as means ± S.D of three replicates/each experimental condition. Values were considered significant for *p* < 0.05, with the indicated superscripts ^*^vs. CTR, ^#^vs. CM_COFZ_, ^§^vs. CTR 2%,^£^vs. T0.

The EMT gene-related profiles were downregulated in cells incubated under 2% O_2_ tension involving *SNAIL, TWIST*, and *VIM* (*p* < 0.05 for both CTR 2% vs. CTR and CM_COFZ_ 2%, vs. CM_COFZ_) (Figure 6A). On the contrary, physoxia induced the upregulation of tendon-related genes: CTR overexpressed *COL I* (*p* < 0.05 CTR vs. CTR 2%) (Figure 6B), whereas CM_COFZ_ 2% overexpressed both the early and late markers, *SCXB* and *TNMD*, respectively (for both *p* < 0.05 CM_COFZ_ 2% vs. CM_COFZ_) (Figure 6B).

The protein quantification of TNMD reinforced the evidence of the stimulatory influence of 2%, O_2_ in promoting tendon differentiation in AECs (Figures 6C,D). Indeed, AECs cultured under physoxia conditions displayed significantly higher levels of TNMD (*p* < 0.05 CTR vs. CTR 2%), reaching values similar to those induced in AECs from CM_COFZ_ exposure (*p* > 0.05 CTR 2% vs. CM_COFZ_ in 21% O_2_). The intracellular TNMD levels were further significantly increased when AECs were exposed to the tendon inductive influence of 2% O2 in combination with the stimulatory action of CM_COFZ_ (*p* < 0.05 CM_COFZ_ 2% vs. CM_COFZ_) (Figures 6C,D).

**Figure 7:**
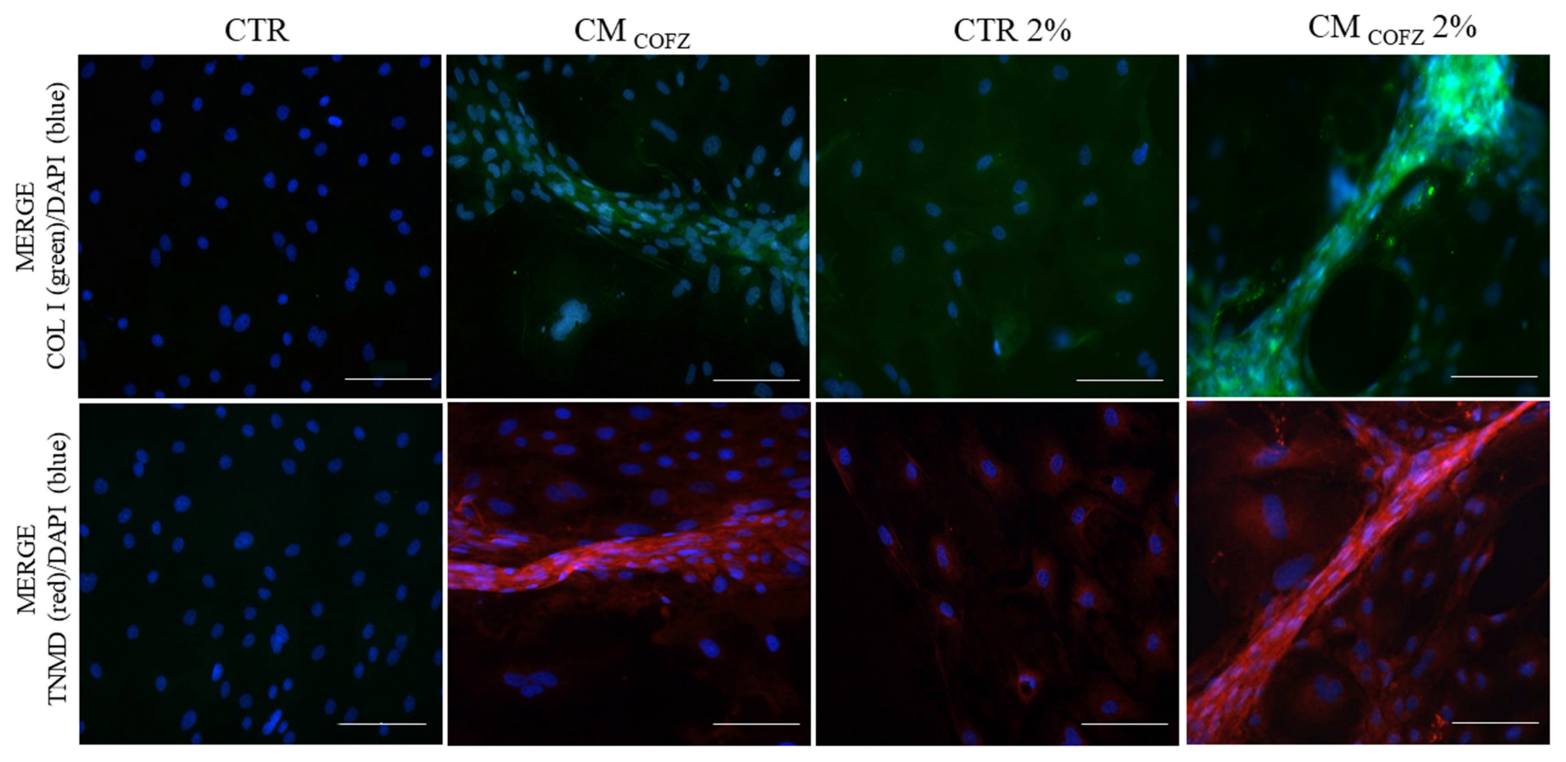
Representative IHC images of COL I (green color) and TNMD (red color) proteins expression in AECs under normoxia and physoxia experimental conditions. Nuclei were counterstained with DAPI (blue color). Magnification 20X, scale bar: 100 μm.

Both COL I (green fluorescence in Figure 7) and TNMD (red fluorescence in Figure 7) proteins were localized in 3D tendon-like structures developed *in vitro* from AECs exposed for 14 days to CM_COFZ_ and CM_COFZ_ 2%. Weak positivity was observed also in CTR AECs incubated under physoxia condition (CTR 2%), independently from their ability to aggregate and develop 3D cell structures (Figure 7).

Altogether, these results suggested that physoxia can enhance the AECs response to the teno-inductive properties of frozen CM_CO_.

### AECs' Alignment Induced by CM_CO_ and CM_COFZ_ 2% Confirmed the Greater Specialization of 3D Tendon-Like Derived Structures

**Figure 8:**
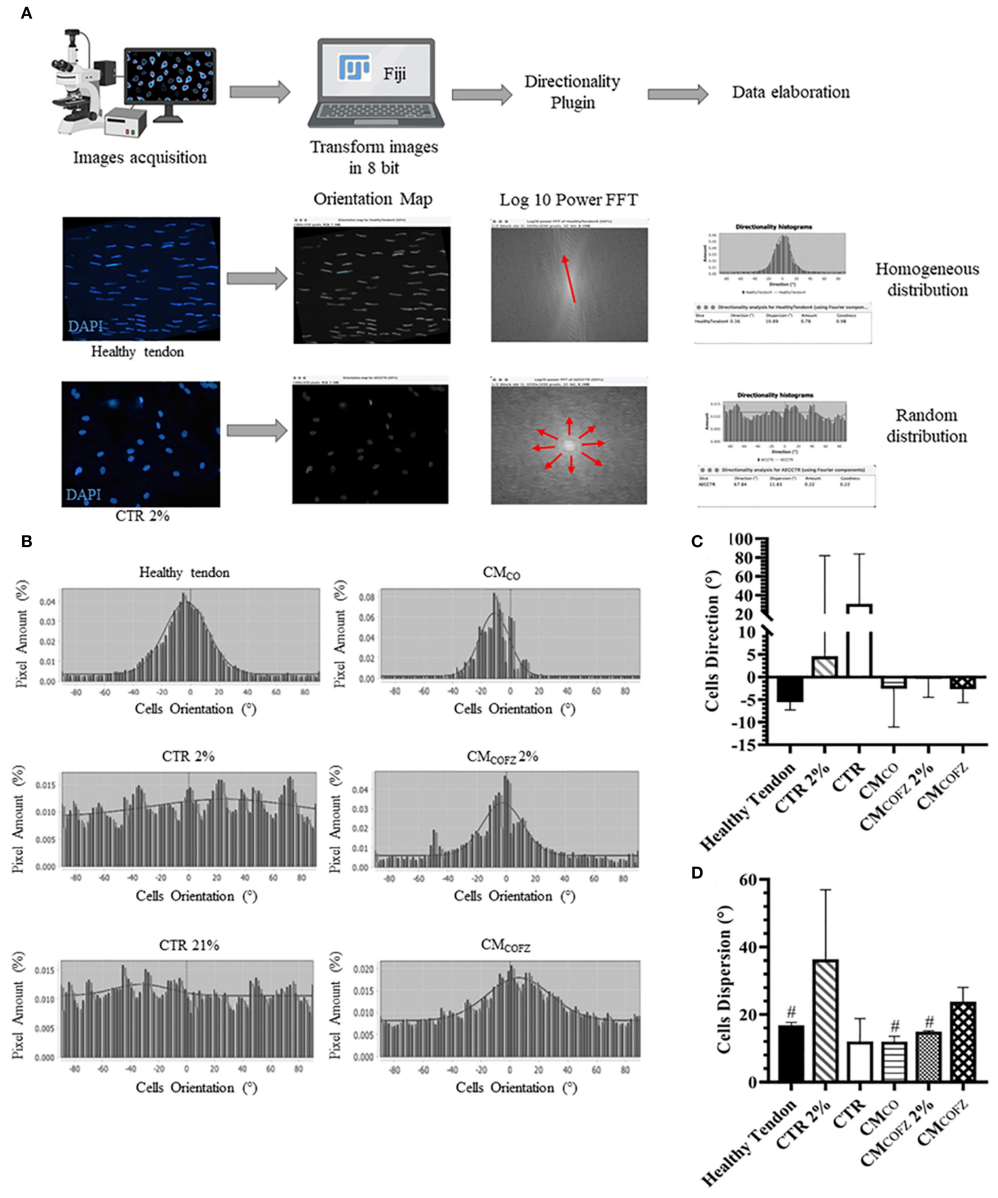
Directionality analyses on AECs cells orientation in 3D tendon-like structures under different culture conditions. **(A)** Representative scheme of the Directionality Plugin used to analyse cells orientation within the different treated groups. **(B)** Representative Gaussian graphs [X = Cells Orientation (°), Y = Pixel Amount (%)]. Analysis of **(C)** angle direction (cells orientation calculated on the bases of the longitudinal axis of the sample) and **(D)** angle dispersion (the standard deviation of the Gaussian). Healthy tendon was used as reference sample for the analyses. Statistical analyses were performed by One Way ANOVA and expressed as means ± S.D of six biological replicates (*n* = 6 animals) for each experimental condition performed in triplicate. Values were considered significant for *p* < 0.05, with the indicated superscript ^#^vs. CM_COFZ_.

CM_CO_ and low levels of O_2_ pressure were also able to impact on the quality of the 3D tendon-like structures developed after 14 days of culture by influencing AECs orientation and alignment (Figure 8). Indeed, according to the Fourier power spectra analyses obtained by the Directionality Plugin of ImageJ schematized in Figure 8A, the qualitative distribution of cell angle direction exposed to CM_CO_, and CM_COFZ_ 2% described a Gaussian curve with a tight shape similar to that recorded in healthy tendon (Figure 8B). On the contrary, both CTR (2 and 21% O_2_) groups developed randomly oriented cells as demonstrated by the pronounced flattening shape of Gaussian curve (Figure 8B). The cells treated with CM_COFZ_ expressed an intermediate behavior (Figure 8B). Even though the global analysis of angle direction did not reveal any significant differences among the groups as a consequence of its great individual variation (Figure 8C, *p* > 0.05), the dispersion values which report the standard deviation of the Gaussian curves confirmed the similarity amongst CM_CO_ and CM_COFZ_ 2% and healthy tendon in respect to CM_COFZ_ (Figure 8D, *p* < 0.05).

## Discussion

This study aims to evaluate the biological effect of CM secretome derived from ovine tendon fetal tissue, in order to propose a new approach able to promote teno-differentiation for veterinary and medical purposes. Tissue co-culture provides a favorable microenvironment for the induction of *in vitro* tissue specific differentiation (Paschos et al., 2015; Xie et al., 2018; Chu et al., 2020). This approach has been successfully applied to *in vitro* tenogenesis (Barboni et al., 2012a), being effective in enabling a stepwise differentiation process in freshly isolated amniotic epithelial cells (AECs). The soluble factors released from tendon explants first drove the freshly isolated AECs toward the mesenchyme phenotype followed by a tendon-like three dimensional organizations which expressed tissue specific markers (Barboni et al., 2012a). Tendon differentiation is represented by a complex biological process characterized by the orchestration of multiple mechanisms including the sequential expression of early and late markers such as SCXB and TNMD, respectively (Schweitzer et al., 2001; Docheva et al., 2005; Dex et al., 2017; Citeroni et al., 2020). In particular, TNMD is generally accepted as structural and functional marker of the mature tendon lineage differentiation (Citeroni et al., 2020). Given its complexity, tenogenesis is hard to achieve, especially *in vitro*, and to date the biological processes and signaling involved are as yet largely unknown. Several teno-inductive techniques are proposed, involving the use of growth factors, biomimetic materials and/or bioreactor, but none of these methods is considered robust in predictably induce a fully committed tendon phenotype (Citeroni et al., 2020). Here, the culture condition for collection of teno-inductive secretomes from tendon explant and AECs co-culture has been defined. These confirmed that teno-induction did not require cell-to-cell interaction, but was achievable through the stimulatory influence of bioactive factors released into the culture media (CM). This provides a powerful strategy for the small scale production of inductive factors sufficient to drive a complete tenogenic differentiation. In addition, AECs proved to be an useful stem cell model for the study of *in vitro* tenogenesis and to test the teno-inductive properties of different stimuli such as different typologies of CM. Starting from an epithelial phenotype, the process of tenogenesis can be documented strictly through the switch on of molecules that are not expressed into freshly derived primary epithelial cells and not from an expression level consistent with a mesenchyme stem cell source (Dai et al., 2015; Ciardulli et al., 2020a). Indeed, the biological response of AECs to CM allowed documentation of distinct bioactivity properties by identifying the greater teno-inductivity of supernatants derived from the co-cultures (CM_CO_) between fetal explants and AECs. Soluble factors accumulated in CM_CO_ displayed a higher ability to promote an AECs phenotype shift through the organization of 3D tendon-like structures that displayed a transcriptional profile characterized by simultaneous EMT and tendon-related gene upregulation (*SNAIL, VIM* and *SCXB, COL III, COL I, TNMD, THSB4*, respectively). Furthermore, the final commitment of AECs toward tendon lineage was strongly confirmed by COL I and TNMD proteins that were largely localized in AECs composing the CM_CO_ derived 3D tendon-like structures in contrast to the AECs incubated under CTR conditions. The teno-inductive factors were released into supernatants of the co-cultures, thus indicating that an active *in vitro* dialogue between AECs and tendon explants was specifically triggered.

Even if soluble factors released in the CM were not characterized, the *in vitro* results showed that an epithelial source of stem cells that does not express any tendon-related protein, were induced to respond to soluble factors released into CM in response to the mutual dialogue between fetal tendon tissue and amniotic derived cells.

The differentiation process induced in AEC had been repeated by reaching consistently tendon lineage genome and protein end point. The biological efficacy of CM balanced out the absence of information about the molecular composition of these secretomes that will required time in order to identified the cocktail of factors involved in an animal model such as sheep, where the molecular codification reached to date is very limited. This is complicated by the large molecular classes that have been described to be involved in teno-induction belonging to lipid, protein and miRNA classes (Citeroni et al., 2020).

Previously *in vitro* and *in vivo* evidences clearly supported the ability of AECs in receiving tenogenic inductive information in response to specific stimuli coming from co-culture with tenocyte of different species or from injured host tendons (Muttini et al., 2018). This information was obtained by adopting different *in vivo* approaches by transplanting ovine AEC under either allograft or xenograft settings (Barboni et al., 2012b; Muttini et al., 2013, 2015, 2018; Mauro et al., 2016).

The xenotransplantation *in vivo* model open, in addition, the scenario to use the ovine derived secretomes also for inducing tenogenesis in other mammals' species. Indeed, by analyzing the genome response of human AEC transplanted into an ovine injured, this concept was confirmed since human AECs xenotransplanted actively dialogued with the host tissue by upregulating several genes involved, at the same time, in tendon regeneration, angiogenesis, and immune response (Barboni et al., 2018).

Moreover, these results strongly suggest that CM secretome could be positively considered in trans-species translational medicine.

On the contrary, CM collected exclusively from fetal tendon explants alone (CM_FT_), were more effective in inducing EMT than tenogenesis. This conclusion is supported by the overexpression by AECs of EMT transcription factor gene *TWIST* and the mesenchyme final marker *VIM*, as well as by their failure to upregulate *TNMD*. Not surprisingly, AECs exposed to CM_FT_ developed a very limited number of 3D tendon-like structures at the end of incubation when compared to CO and CM_CO_. Moreover, TNMD protein levels, detected by Western Blot analysis, were significantly reduced respect to CO and CM_CO_. These results indicated that tendon explants alone were not able to generate *per se* factors able to recapitulate a complete tenogenic differentiation, whereas they release bioactive molecules driving EMT as a culture end point.

In the absence of validated markers indicative of tenogenesis, in the present study teno-induction was confirmed by complementarity of gene, protein and morphometric data. The gene and protein profile are relevant, since both early and late tendon-related genes were low or absent in AECs cultured in the absence of any inductive stimulus. The morphological/ morphometric analysis of AECs revealed that tendon-like structures were achieved in response to an active process driven by CO or by the AECs exposure to CM_CO_. On the contrary, AECs under CTR condition did not aggregate or align in 3D structures, instead maintaining the native epithelial phenotype. These results defined, for the first time, the protocol for small-scale production of teno-inductive soluble factors while simultaneously validating *in vitro* AECs culture as a tool for bioactivity assay.

The availability of tendon-inductive factors may have research and application impacts in both helping establish robust *in vitro* tendon differentiation protocols and facilitating the development of tendon tissue engineering approaches. The availability of bioactive CM with defined teno-inductive properties may be applied to either scaffold functionalisation (Burdette et al., 2018; Chen et al., 2021) or in nanoparticle generation (Felice et al., 2018), both of which may have a clinically relevant role in the development of a new therapies for tendon pathology (Daneshmandi et al., 2020; Rhatomy et al., 2020).

Tissue engineering protocols directed toward tendinopathies (Andia and Maffulli, 2019) will require that the CM_CO_ secretome drive signaling in a low oxygen environment which, in tendons, is physiological. In fact, tendon exists in a low oxygen milieu given the poor vascularisation of the tissue (Benjamin and Ralphs, 1997). While skeletal muscle oxygenation is estimated to be 2–5% O_2_, the oxygen consumption of tendons and ligaments is likely 7.5 times lower (Sharma and Maffulli, 2005). This means that tenocytes live in a physiological low O_2_ environment (Shukunami et al., 2008). Low oxygen pressure is required, alongside specific growth factors, to induce tendon differentiation in embryonic stem cells (Dale et al., 2018) and, in combination with others stimuli, in mesenchymal stem cells (Yu et al., 2016; Zheng et al., 2017). Regarding this particular aspect, the present study demonstrated, using AECs, that low oxygen (physoxia, 2% O_2_) is a permissive condition enabling AECs to EMT. Low oxygen has been established as a regulator of EMT in pathological processes such as tumorigenesis (Chen and Wu, 2016) of breast (De Francesco et al., 2018), prostate (Tang et al., 2019), and lung cancer (Kohnoh et al., 2016), or in mesothelial cells triggering peritoneal fibrosis formation (Morishita et al., 2016). However, EMT is a biological process associated not only with pathological conditions, but also with physiological processes which take place during embryonic development, morphogenesis and stem cells differentiation (Chen et al., 2017). Moreover, EMT was recently demonstrated to be involved in Achilles tendon repair in rats model (Sugg et al., 2014). Intriguingly, the present results demonstrated that physoxia can be exploited to overcome the negative effect of freezing on the teno-inductivity of CM_COFZ_. Working upwards from a baseline principle that CM_CO_ storage is an unavoidable procedure to allow its future, off-the-shelf, widespread application, the effect of freezing has been analyzed. Storage at −80°C partially affected the teno-inductive potential of CM_CO_. Indeed, despite no differences in *SCXB* and *COL I* mRNA content, the expression of the late tendon marker *TNMD* was significantly decreased in AECs exposed to CM_COFZ_ with respect to CM_CO_, even though its level was maintained over CTR. These data suggested that CM_COFZ_ was able to start the tenogenic process in AECs, but was unable to achieve the differentiation end point. Indeed, CM_COFZ_ resulted in a reduced formation of 3D tendon-like structures which, however, were positive for COL I and TNMD protein expression. Conversely, CM_COFZ_ when combined with a physoxic environment enhanced AECs differentiation and stimulated elevated expression of early, *SCXB*, and late TNMD, markers at both mRNA and protein levels. Biological data (gene expression and protein distribution) combined with morphological results, obtained in AECs exposed to CM_COFZ_ under physoxia, suggested that cryogenic storage did not switch off the teno-inductive molecules secreted but, it could modify their availability and or/concentration. Physoxic culture could enhanced the capacity of AECs to respond to the molecular tenogenic factors present in frozen CM_CO_, as demonstrated by the 3D structures that reached morphological and biochemical levels similar to those recorded in healthy tendons and in CM_CO_ treatment. Moreover, directionality analysis confirmed the differences in cells angle dispersion between CM_CO_ and CM_COFZ_ 2% respect to CM_COFZ_. The hypothesis that low oxygen can be the most suitable culture condition to induce tenogenesis, is also supported by TNMD upregulation in AECs under physoxic condition (CTR 2%) observed in this study. According to our results, enhanced *TNMD* expression was demonstrated in human embryonic stem cells (hESCs) under low oxygen culture without any further stimulation (Dale et al., 2018). The Authors indicated that 2% O_2_ supplemented with a cocktail of bone morphogenetic protein-12,−13 (BMP-12, BMP-13), and ascorbic acid (AA) represented the best culture system to induce tenogenesis in hESCs (Dale et al., 2018). According to this literature data, physoxia, the natural environment of stem cells niche (Mohyeldin et al., 2010) and also of amniotic stem cells (Johnell et al., 1971; Banerjee et al., 2018), can enhance tendon differentiation biological response as demonstrated for adipose-derived mesenchymal stem cells (Yu et al., 2016) and for menstrual blood stromal stem cells (Zheng et al., 2017).

Even if, in the absence of a complete molecular characterization and without any evidence supporting the cell-to cell communication mechanisms involved, several evidences confirmed the biological role of CM derived from amniotic cells under *in vitro* and *in vivo* conditions (Rossi et al., 2012; Lange-Consiglio et al., 2013a). More in detail, the CM-derived from amniotic stem cells were recently used *in vivo* to improve clinical recovery in spontaneous tendinopathies in 13 horses (Lange-Consiglio et al., 2013a). This manuscript confirmed the idea that, even in the absence of any molecular characterization, the strong biological outcome obtained can be indirectly used to confirm that AEC may have a positive role in inducing tendon regeneration adopting a paracrine action (Barboni et al., 2012b, 2018; Lange-Consiglio et al., 2013b; Muttini et al., 2013, 2015, 2018). However, any practical application of such cells derivates, however, pass through the possibility to preserve their biological influence unaltered over time.

Long term storage technologies of bio-derivate compounds are available and could be tested under strict experimental conditions to evaluate their efficacy. Lyophilisation removes water from frozen samples by sublimation and desorption in a vacuum (Chen et al., 2010), and it is used to preserve biological materials such as proteins (Roy and Gupta, 2004; Jain et al., 2019), plasma (Jennings et al., 2015; Storch et al., 2019), and also living cells (Keskintepe and Eroglu, 2015). Moreover, lyophilisation improved the long-term stability of nanosized drug delivery, such as liposomes (Liu et al., 2015) and exosomes (Charoenviriyakul et al., 2018). It was also demonstrated as a solution to preserve the whole secretome (Fernandes-Cunha et al., 2019; Chen et al., 2021).

Lyophilisation, even though not been performed yet in this setting, could be explored for utility in preservation of CM_CO_. There could be several advantages of the use of tenogenic secretome obtained with this strategy in the field of regenerative medicine. The use of the CM secretome has advantages over the implantation of the stem cells themselves, as their components, such as extracellular vesicles, liposomes, growth factors, and miRNA can be bioengineered and scaled to specific dosages, and the cell-free nature of the secretome enables it to be efficiently stored and transported. The CM_CO_ secretome could be used to produce enriched nanodevices for tendon tissue regeneration. According to the use of secretome for tissue regeneration, Felice et al. (2018) demonstrated that nanoparticles loaded with endothelial progenitor cell (EPC) secretome contributed to ischemic tissue repair by controlled paracrine secretion upregulated by hypoxia. Secretome charged nanoparticles could be advantageous as the technique allows close spatiotemporal control on the kinetic release of the CM (Felice et al., 2018), and could be used to directly inject local focal lesions or to functionalise scaffolds (Tang et al., 2017; Shoma Suresh et al., 2020; Chen et al., 2021).

The composition of nanoparticles themselves is to be taken into account to elaborate appropriate storage conditions, and it must be functional to the intended use. Furthermore, both nanovescicles or nanobeads can be chosen to reach this goals, and both can ensure a controlled or targeted delivery of their payload, respectively, as recently suggested (Ciaglia et al., 2019; Palazzo et al., 2021). On the other hand, the main component of the CM has to be investigated to study the kinetic of the release (Felice et al., 2018).

In the future, the characterization of CM_CO_ secretome will be pursued to identify which molecular combination is involved in inducing tenogenesis.

This will be a great attainment either to solve an open challenges of veterinary and medicine tissue engineering or to take advantage of nanomedicine technologies (pharmacokinetic studies and storage protocols) to use teno-inductive secretomes for scaffolds functionalisation.

Even though an epithelial stem cell source was used as proof of concept to confirm the teno-inductive properties of CM_CO_ before proposing them as a novel therapeutic biological cell-free product to support tendon regeneration, their validation on other stem cell sources derived from species of vet and medicine relevance or on *in vivo* settings have to be considered before moving their use toward clinical applications.

## Conclusion

In conclusion, the major goal of the present study was the definition of an *in vitro* protocol for the collection of bioactive teno-inductive factors (CM_CO_) and to preserve them over time. A small scale production of validated teno-inductive supernatants may have a great impact for research and for developing TE to restore tendon microarchitecture and function. Indeed, the proposed *in vitro* system that models tenogenesis by recapitulating the native paracrine phenotypic patterns may result in significant advances in tendon biology and tenogenic phenomena at the cellular and molecular levels. At the same time, the availability of low-cost bioactive factors with assayable teno-inductive properties in combination with the progress of biomedical technologies (nanomedicine and scaffold design and fabrications) may provide practical answer and sustainable solution to establish cells free therapies protocols by innovating the challenging field of tendon medicine.

## Data Availability Statement

The original contributions presented in the study are included in the article/supplementary material, further inquiries can be directed to the corresponding authors.

## Author Contributions

BB conceptualized whole the research. MRC performed cell culture, RT-qPCR and statistical data analyses. AM supervised data analyses, and revised the manuscript. VR performed IHC analysis, MD performed Western Blot analysis. ME performed cells orientation analyses. MT performed stem cells isolation and characterization. NF designed physoxia experiments and revised the manuscript. MCC and MS performed physoxia culture experiments. BB and MRC wrote and edited the manuscript. GD and NM revised the manuscript. BB provided research grants. All authors validated the data and reviewed the manuscript and have read and agreed to the published version of the manuscript.

## Conflict of Interest

The authors declare that the research was conducted in the absence of any commercial or financial relationships that could be construed as a potential conflict of interest.
